# A Combined Flow Cytometric Semen Analysis and miRNA Profiling as a Tool to Discriminate Between High- and Low-Fertility Bulls

**DOI:** 10.3389/fvets.2021.703101

**Published:** 2021-07-19

**Authors:** Federica Turri, Emanuele Capra, Barbara Lazzari, Paola Cremonesi, Alessandra Stella, Flavia Pizzi

**Affiliations:** Institute of Agricultural Biology and Biotechnology, National Research Council (IBBA-CNR), Lodi, Italy

**Keywords:** bull, sperm, semen quality, sequencing, miRNA, fertility

## Abstract

Predicting bull fertility is one of the main challenges for the dairy breeding industry and artificial insemination (AI) centers. Semen evaluation performed in the AI center is not fully reliable to determine the level of bull fertility. Spermatozoa are rich in active miRNA. Specific sperm-borne miRNAs can be linked to fertility. The aim of our study is to propose a combined flow cytometric analysis and miRNA profiling of semen bulls with different fertility to identify markers that can be potentially used for the prediction of field fertility. Sperm functions were analyzed in frozen-thawed semen doses (CG: control group) and high-quality sperm (HQS) fraction collected from bulls with different field fertility levels (estimated relative conception rate or ERCR) by using advanced techniques, such as the computer-assisted semen analysis system, flow cytometry, and small RNA-sequencing. Fertility groups differ for total and progressive motility and in the abnormality degree of the chromatin structure (*P* < 0.05). A backward, stepwise, multiple regression analysis was applied to define a model with high relation between *in vivo* (e.g., ERCR) and *in vitro* (i.e., semen quality and DE-miRNA) fertility data. The analysis produced two models that accounted for more than 78% of the variation of ERCR (CG: *R*^2^ = 0.88; HQS: *R*^2^ = 0.78), identifying a suitable combination of parameters useful to predict bull fertility. The predictive equation on CG samples included eight variables: four kinetic parameters and four DNA integrity indicators. For the HQS fraction, the predictive equation included five variables: three kinetic parameters and two DNA integrity indicators. A significant relationship was observed between real and predicted fertility in CG (*R*^2^ = 0.88) and HQS fraction (*R*^2^ = 0.82). We identified 15 differentially expressed miRNAs between high- and low-fertility bulls, nine of which are known (miR-2285n, miR-378, miR-423-3p, miR-191, miR-2904, miR-378c, miR-431, miR-486, miR-2478) while the remaining are novel. The multidimensional preference analysis model partially separates bulls according to their fertility, clustering three semen quality variable groups relative to motility, DNA integrity, and viability. A positive association between field fertility, semen quality parameters, and specific miRNAs was revealed. The integrated approach could provide a model for bull selection in AI centers, increasing the reproductive efficiency of livestock.

## Introduction

Prediction of bull fertility is one of the crucial factors dictating optimum efficiency in the livestock production system. Considering that, in dairy cattle, the selection and reproductive management are based on the use of artificial insemination (AI), the assessment of the level of bull fertility nowadays is one of the main challenges for AI centers and cattle breeders.

At present, quality parameters routinely applied in semen evaluation tests performed in AI centers are not fully reliable to determine a bull's fertility level. Field fertility, in terms of the ability of bulls to make cows pregnant through AI, is affected by a wide range of factors, such as health status, genetic traits, full functionality of reproductive organs, herd management, semen quality, and cryopreservation protocols. Reproductive efficiency of AI bulls is predicted by direct measure of fertility. The most utilized method of measuring male fertility is related to the non-return rate (NRR) although low-to-moderate correlation has been observed between the semen quality criteria currently used and the NRR (1). The estimated relative conception rate (ERCR) is a measure of the fertility of an individual sire and is predictable and repeatable over the whole productive life of an AI sire if data relative to the semen quality have been collected (2).

Studies on male fertility focus also on the assessment of several semen quality parameters after freezing and thawing, on the evaluation of the relationship of *in vitro* semen quality with field data, with the aim to exclude low-potential-fertility bulls from breeding programs, thus saving considerable time and economic resources. As genomic selection for male fertility is not widely implemented, semen evaluation for the prediction of male fertility has a greater impact on breeding outcomes (3). Thus, an urgent need to improve semen evaluation techniques and fertility prediction models is evident.

Several advanced technologies can be used to examine the quality of spermatozoa—such as computer-assisted semen analysis (CASA) and flow cytometry (FCM)—which can provide accurate and objective evaluation of sperm function. With the existing methods, it is easier to identify low-fertility (LF) bulls, but ranking those with moderate-to-high fertility is more complicated (1). Some researchers, such as Sellem et al. (4), Gliozzi et al. (5), and Kumaresan et al. (6), aimed to identify the most suitable spermatic parameters for semen fertility prediction, combining several *in vitro* assessments and obtaining an accuracy of the developed model for male fertility prediction in cattle species of *R*^2^ = 0.39, *R*^2^ = 0.84, and *R*^2^ = 0.83, respectively. These studies demonstrate that the combination of kinetic semen parameters and DNA analysis based on FCM, such as DNA sperm integrity, seems to be able to distinguish among fertility levels in bulls even in the high-fertility (HF) range.

Moreover, the integration of several tests, from the evaluation of semen quality with advanced technologies to sperm molecular investigation, such as small RNA profiling, is a promising approach to achieve a better understanding of sperm functions as well as to evaluate semen quality and predict bull fertility (7).

Mature spermatozoa contain coding and non-coding RNAs, some of which are involved in the regulation of many physiological pathways related to spermatogenesis, sperm maturation, and early embryonic development with the potential to modulate sperm morpho-functional features as well as male reproductive ability (8). Among small RNA species, miRNAs are associated with sperm cell fertility status and embryo development (9). In bulls, different profiles of sperm miRNA were investigated and associated with different sperm quality and fertility levels (1, 4, 7, 8, 10).

In our study, we hypothesize that bulls with moderate-to-high fertility can be identified by a difference in semen quality and by the analysis of miRNA expression.

An integrated approach between advanced semen quality analysis and miRNA profiling of cryopreserved semen in HF and LF bulls is proposed to identify markers and build a model that can be potentially used for the prediction of male field fertility.

## Materials and Methods

### Semen Source and Field Fertility Evaluation

Frozen semen samples from 10 mature progeny-tested Italian Holstein–Friesian bulls of known reproductive ability were purchased from INSEME S.p.A bull AI center (Modena, Italy). The bulls were classified according to field fertility data expressed as the ERCR, provided by the Italian Holstein Breeders Association (ANAFI). To calculate the ERCR, the 56-day NRR to service was adjusted for several factors, including farm, year and month of insemination, parity, days open of the cow at first insemination, bull, bull progeny, and cow. The ERCR represents the effect of the bull on the NRR (expressed as a percentage) of cows inseminated in the herd and is determined by the difference between that NRR value for a particular bull and the average NRR values obtained with the semen of other bulls. As its calculation is based on a large number of services from many different herds, the ERCR is considered a highly accurate measurement to identify HF and LF bulls (11). On average, 1,541 inseminations per bull (with a range of 106–11,036 inseminations) were used to estimate ERCR. Bulls with a reliability of ERCR > 80 were considered (Reliability = 100 × [1 – (1 – (number of inseminations / (number of inseminations + 200))½) × 2.3]). According to the distribution of bulls based on the ERCR, five HF and five LF bulls were identified in the distribution tails. The bulls included in the present study, routinely used in AI, displayed a narrow fertility range, as ERCR values ranged from −2.09 to 4.09 (Table 1). For each bull, two different batches were collected in two seasons, summer (August–September) and winter (February–March) and analyzed. The semen was packaged in 0.5-ml straws (each containing 20 × 10^6^ spermatozoa), frozen with a commercial egg yolk–based extender, and stored in liquid nitrogen. For each bull, the frozen-thawed semen doses were evaluated before (control group: CG) and immediately after Percoll density gradient centrifugation (high-quality sperm, HQS, fraction).

**Table 1:** Field fertility details of the bulls involved in the study.

**Bulls**	**ERCR**	**Fertility**	**N. of insemination**	**Reliability (%)**
1	2.38	High	4,202	99
2	1.97	High	3,209	98
3	4.09	High	2,371	98
4	1.99	High	4,074	99
5	2.84	High	16,767	99
6	−2.41	Low	346	85
7	−1.73	Low	1,892	97
8	−2.90	Low	1,427	96
9	−1.82	Low	2,929	98
10	−1.83	Low	360	86

### Isolation of Spermatozoa Through Percoll Gradient

HQS fractions were isolated as described by Capra et al. (7). For each bull, 10 frozen semen doses (20 × 10^6^ cells per dose) were simultaneously thawed in a water bath at 37°C for 20 s and pooled. The pool (5 mL) of each bull was split into two aliquots of 2.5 mL that were overlaid on a dual-layer (90–45%) discontinuous Percoll gradient (Sigma-Aldrich, St. Louis, USA) in two 15-ml conical tubes and centrifuged at 700 × *g* for 30 min at 20°C. The Percoll layers were prepared by diluting Percoll solution in as indicated by Parrish et al. (12). The Percoll gradient is a colloidal suspension of silica particles coated with polyvinylpyrrolidone. By using two discontinuous layers (45 and 90%) by centrifugation it is possible to obtain a different sedimentation according to sperm motion. The HQS fraction obtained from each of the two tubes (replicates) was washed in Tyrode's albumin lactate pyruvate (TALP) sperm medium (12) at 700 × *g* for 10 min at 20°C; the obtained pellets were resuspended in 150 μl of TALP. Aliquots from each replicate were kept at −80°C until RNA extraction.

### Evaluation of Sperm Characteristics

Two technical replicates for each bull, for the two seasons, were evaluated for total motility and sperm kinetic parameters by CASA and sperm viability, acrosomal status, and DNA integrity by FCM immediately after thawing (CG) and after Percoll separation in the HQS fraction obtained.

#### CASA

Total motility and sperm kinetics parameters were assessed by the CASA system (ISAS^®^ v1, Proiser, R + D S.L., Paterna, Spain) combined with a phase contrast microscope (Nikon Optiphot) equipped with a negative phase contrast 10 × objective and integrated warmer stage and connected to a video camera (Proiser 782M, Proiser R+D).

Semen from CG was evaluated as raw without dilution, and 5 μl of semen pellet obtained after Percoll density gradient centrifugation (HQS fraction) were diluted in 5 μl TALP sperm medium (13) prewarmed at 37°C. Next, 10 μl of diluted semen were placed on a prewarmed (37°C) Makler^®^ chamber. During the analysis, the microscope heating stage was maintained at 37°C. Images were relayed, digitized, and analyzed by the ISAS^®^ v1 software with user-defined settings as follows: frames acquired, 25; frame rate, 20 Hz; minimum particle area 20 microns^2^; maximum particle areas 70 microns^2^; progressivity of the straightness 70%. CASA kinetics parameters were total motility (MOT TOT, %), progressive motility (PRG, %), curvilinear velocity (VCL, μm/s), straight-line velocity (VSL, μm/s), average path velocity (VAP, μm/s), linearity coefficient (LIN, %, = VSL/VCL × 100), amplitude of lateral head displacement (ALH, μm), straightness coefficient (STR, % = VSL/VAP × 100), wobble coefficient (WOB, % = VAP/VCL × 100), and beat cross frequency (BCF, Hz).

#### FCM Analysis

Measurements were recorded using a Guava^®^ EasyCyte™ 5HT microcapillary flow cytometer (Merck KGaA, Darmstadt, Germany; distributed by IMV Technologies) equipped with fluorescent probes excited by a 20-mW argon ion laser (488 nm). Forward-scatter (FSC) vs. side-scatter (SSC) plots were used to separate sperm cells from debris. Non-sperm events were excluded from further analysis. Fluorescence detection was set with three photomultiplier tubes: detector FL-1 (green: 525/30 nm), detector FL-2 (yellow/orange: 586/26 nm), and detector FL-3 (red: 690/50 nm). Calibration was carried out using standard beads (Guava^®^ Easy Check Kit, Merck Millipore). A total of 5,000 sperm events per sample for each bull were analyzed at a flow rate of 200 cells/s. Compensation for spectra overlap between fluorochromes was set according to the procedures outlined by Roederer (14).

Data were acquired and analyzed using CytoSoft and EasyCompDNA software (Merck KGaA, Darmstadt, Germany; distributed by IMV Technologies), respectively.

##### Sperm Viability

The LIVE-DEAD^®^ Sperm Viability Kit (Life Technologies Italia, Italy) was used for the analysis of plasma membrane integrity as described by Gliozzi et al. (5). The kit contained the membrane-permeant nucleic acid stain SYBR^®^14 and the conventional dead cell stain propidium iodide (PI). Both dyes can be used to label DNA. After staining, live sperm cells with intact cell membranes fluoresced bright green, whereas cells with damaged cell membranes fluoresced red. Aliquots of semen extended with Easy Buffer (2.0 × 10^5^ spermatozoa/ml) were supplemented with SYBR^®^14 0.1 μM and PI 12 μM (final dilutions) according to manufacturer instructions. After gentle mixing and 10 min incubation at 37°C in the dark, three replicates per sample were performed. Debris particles were gated out according to the intensity of green and red fluorescence. Three sperm populations were detected on the FL-1/FL-3 dot plot: viable (green), dead (red), and moribund (double-stained) spermatozoa.

##### Acrosomal Membrane Integrity

Acrosomal membrane integrity was assessed using Easykit 5 (ref. 025293; IMV Technologies). This kit is used to measure the level of disrupted acrosome within viable or dead sperm populations. The ready-to-use 96-well plate was filled with 200 mL of embryonic holding solution (ref. 019449; IMV Technologies), and 40,000 sperm were added during 45 min at 37°C protected from light in basal condition. The signal was read with the Guava^®^ EasyCyte^™^ 5HT microcapillary flow cytometer, and a total of 5,000 events were acquired. Percentages of sperm with either intact or damaged plasma membrane as well as intact or damaged acrosome were computed. Spermatozoa with disrupted acrosomes were labeled with a green probe. Dead spermatozoa with damaged plasma membrane were labeled with a red fluorochrome.

##### Sperm Chromatin Structure Assay

The Sperm Chromatin Structure Assay was assessed as previously described in other studies of ours (5, 15) by using acridine orange, a planar molecule that intercalates into double-stranded DNA but stacks on single-stranded DNA causing a metachromatic shift from green (double-stranded DNA) to red fluorescence (single-stranded DNA), when exposed to the 488 nm laser light of the flow cytometer. The technique is based on the susceptibility of sperm DNA to acid-induced denaturation as low pH treatment causes partial DNA denaturation in sperm with altered chromatin structure. The assessments were performed using the sperm chromatin structure assay (16): 3.0 × 10^5^ cells were diluted in 200 μl of TNE buffer (0.01 M Tris-HCl, 0.15 M NaCl, 1 mM EDTA, pH 7.4) and added to 400 μl of an acidic solution (Triton X-100 0.1%, 0.15 M NaCl, 0.08 N HCl; pH 1.2). After 30 s, cells were stained with 1.2 ml of acridine orange solution (6 μg/ml in 0.1 M citric acid, 0.2 M Na_2_HPO_4_, 1 mM EDTA, 0.15 M NaCl; pH 6). Samples were protected from light and incubated at room temperature for 2.5 min; after that, two replicates per sample were acquired and analyzed using, respectively, CytoSoft and EasyCompDNA software (Merck KGaA, Darmstadt, Germany; distributed by IMV Technologies) and expressed as Alpha-T, which is indicative of the shift from green to red fluorescence, is expressed as the ratio of red to total fluorescence intensity [red/(red + green)], and quantifies the degree of abnormal chromatin structure with an increased susceptibility to acid-induced denaturation; standard deviation of Alpha-T (ATSD), which shows the extent of abnormality in the chromatin structure within a population; DNA fragmentation index (%DFI), which indicates the percentage of sperm with fragmented DNA; and percentage of sperm with high green fluorescence (%HG), which is representative of the percentage of immature cells with reduced nuclear condensation (incomplete histone-protamine exchange). The FCM instrument was AO-saturated prior to analysis by running the AO equilibration solution.

### RNA Extraction

Two technical replicates of the HQS Percoll fraction (obtained from 10 frozen semen doses each) from each bull sperm (*n* = 10) collected in two seasons (summer and winter) were used for RNA isolation. RNA was extracted as previously described (7). Briefly, the sperm cell pellet was initially homogenized and solubilized in 800 μl of TRIzol with incubation at 65°C for 15 min. RNA was then purified using the NucleoSpin^®^miRNA kit (Macherey-Nagel, Germany). RNA concentration and quality were determined by the Agilent 2100 Bioanalyzer (Santa Clara, CA) (Supplementary File 1). The isolated RNAs were stored at −80°C until use.

### Library Preparation and Sequencing

Libraries were generated using the Illumina Truseq Small RNA Preparation kit according to the manufacturer's instructions and the protocol described by Capra et al. (7). Libraries from the 10 bulls (five HF, five LF) in the two seasons (winter and summer) were pooled together for each technical replicate and concentrated (15 × libraries pool) with Agencourt^®^AMPure^®^ XP (Beckman, Coulter, Brea, CA) (1 Vol. sample: 1.8 Vol. beads), thus obtaining two pools of libraries representing the two technical replicates. The two pools were purified on a Pippin Prep system (Sage Science, MA, USA) to recover the 125–167 nt fraction containing mature miRNAs. The quality and yield after sample preparation were measured with an Agilent 2200 Tape Station, High Sensitivity D1000. Libraries were sequenced on two lanes of Illumina Hiseq 2000 (San Diego, CA).

### miRNA Detection and Bioinformatics Analysis

miRNA detection and discovery were carried out with Mirdeep2 on Illumina high-quality trimmed sequences. *Bos taurus* miRNAs available at miRBase (http://www.mirbase.org/) were used to accomplish known miRNA detection on the trimmed sequences. Known miRNAs from related species (sheep, goat, and horse) available at miRBase were also input into Mirdeep2 to support the individuation of novel miRNAs. The Mirdeep2 quantifier module was used to quantify expression and retrieve counts for the detected known and novel miRNAs. Differential expression analyses between the HF and LF bulls and in different seasons were run with the Bioconductor edgeR package (17). Principal component analysis (PCA) was performed with Genesis (18).

### Statistical Analysis

Data obtained from *in vitro* semen evaluation (CASA and FCM measurements) were analyzed using the SAS^™^ package v 9.4 (SAS Institute Inc., Cary, NC, USA) with different statistical models to evaluate the following:

1. Isolation of sperm fractions and evaluation of sperm separation.
  The general linear model procedure (PROC GLM) was used to (i) evaluate the effect of technical replicates on semen quality parameters in the HQS fraction—the model included as fixed effects the bull and the replicate nested in the sperm fraction—and (ii) verify the efficiency of the sperm separation from the CG to HQS fractions. The model included the fixed effect of the sperm fraction and of the bull.
2. Relations between *in vitro* semen quality and *in vivo* fertility.
  PROC GLM was used to assess *in vitro* sperm quality in function of the ERCR (HF and LF bulls). The model included the fixed effects of fertility rank, categorized as LF (ERCR ≤ 1.0) and HF (ERCR ≥ 1) and season of semen collection (two levels as follows: winter, February to March, and summer, August to September).

A backward stepwise multiple regression analysis (PROC REG) was used to obtain a model with high relation between sperm quality parameters and ERCR. The redundant parameters were excluded from analysis. The procedure started with a model that contained all *in vitro* sperm parameters and repeatedly eliminated non-significant variables, using a significance level of *P* < 0.05 to retain variables. The real values of the significant variables obtained from each bull as determined by backward elimination were included in an equation (real values were multiplied for the estimated parameters, calculated by backward elimination) to calculate the predicted fertility values. Correlation between real and predicted fertility values was then assessed.

A multidimensional preference analysis (MDPREF) was then conducted to explore the *in vitro* semen quality traits and miRNA expression that correlated with the level of bull fertility. MDPREF is a PCA of data performed using the PROC PRINQUAL procedure of SAS (19). This procedure finds linear and non-linear transformations of variables, using the method of alternating least squares, which optimizes properties of the transformed variables' correlation or covariance matrix. In the current case, a MDPREF biplot with bull fertility level (HF and LF) in rows and *in vitro* semen quality traits and miRNA expression in columns best represents the relationships among the fertility scores. MDPREF identifies the variability that is most salient to the preference patterns of the fertility levels toward the *in vitro* semen quality and miRNAs expression and extracts this as the first principal component.

Results are given as adjusted least squares means ± standard error means (LSM ± SEM).

## Results

### Isolation of Sperm Fractions and Evaluation of Sperm Separation

No significant differences were observed among technical replicates concerning semen quality parameters in HQS fractions (Supplementary Table 1). Sperm cells were successfully enriched in HQS fractions (Table 2) after Percoll centrifugation considering MOT TOT, PROG, VSL, VCL, VAP, ALH, VIAB, VIA, VDA, Alpha-T, and %DFI variables, and a significant (*P* < 0.05) improvement of the sperm quality occurred in the HQS fraction with respect to the CG.

**Table 2:** Sperm quality variables assessed after thawing (Control Group = CG) and after Percoll separation in High and Low Quality Sperm Fraction (HQS, LQS).

**Sperm parameters**	**CG**	**HQS Fraction**	**LQS Fraction**
MOT TOT	73.14 ± 2.51^a^	81.02 ± 2.51^b^	11.15 ± 2.51^c^
PROG	49.15 ± 2.03^a^	52.49 ± 2.03^a^	7.84 ± 2.03^b^
VSL	53.29 ± 2.39^a^	62.80 ± 2.39^b^	42.24 ± 2.39^c^
VCL	86.26 ± 3.59^a^	112.27 ± 3.59^b^	66.78 ± 3.59^c^
VAP	61.94 ± 2.65^a^	75.98 ± 2.65^b^	47.02 ± 2.65^c^
LIN	62.06 ± 1.77^a^	55.25 ± 1.77^b^	63.05 ± 1.77^c^
STR	85.91 ± 0.98^a^	81.81 ± 0.98^b^	88.69 ± 0.98^c^
WOB	72.01 ± 1.48^a^	67.00 ± 1.48^b^	70.33 ± 1.48^b^
ALH	2.85 ± 0.11^a^	3.67 ± 0.11^b^	2.30 ± 0.11^c^
BCF	10.05 ± 0.34^a^	9.03 ± 0.34b	8.33 ± 0.34^b^
VIAB	44.40 ± 2.14^a^	64.31 ± 2.22^b^	24.23 ± 2.22^c^
VIA	25.10 ± 1.62^a^	39.16 ± 1.68^b^	8.56 ± 1.68^c^
VDA	1.96 ± 0.14^a^	1.60 ± 0.15^a^	1.00 ± 0.15^b^
ALPHAT	0.4123 ± 0.001147^a^	0.4072 ± 0.001147^b^	0.4060 ± 0.001183^b^
ATSD	0.0102 ± 0.000597^ab^	0.0118 ± 0.000597^b^	0.0132 ± 0.000616^c^
%DFI	1.60 ± 0.22^a^	0.92 ± 0.22^b^	1.82 ± 0.23^a^
%HG	1.84 ± 0.20^a^	2.05 ± 0.20^a^	3.18 ± 0.21^b^

*MOT TOT, total motility; PROG, cells progressive motility; VSL, straight-line velocity; VCL, curvilinear velocity; VAP, average path velocity; LIN, linear coefficient; STR, straightness coefficient; WOB, wobble coefficient; ALH, amplitude of lateral head displacement; BCF, beat cross-frequency; VIAB, viable sperm; VIA, viable with intact acrosome; VDA, viable with disrupted acrosome; ALPHA-T, red/(red+ green) fluorescence intensity; ATSD, Alpha-T standard deviation; %DFI, fragmented DNA sperm; %HG, high green fluorescence sperm*.

a,b,c *values within a row with different superscripts differ significantly at P < 0.05*.

### Existing Relations Between *in vitro* Semen Quality and *in vivo* Fertility

No significant effects on *in vitro* semen quality were observed according to the semen collection season. *In vitro* sperm quality parameters according to the fertility level, assessed on both the CG and HQS fraction, are presented in Table 3. A significant (*P* < 0.05) improvement of MOT TOT and PROG was found in CG/HF bulls with respect to the CG/LF group. Significant differences were also observed for Alpha-T, showing a major abnormality in the degree of chromatin structure in LF bulls. In the CG/HF bulls, higher values were found with respect to LF bulls for VIAB and VIA variables as well as a lower percentage of sperm with fragmented DNA (%DFI) and percentage of sperm with immature cells with reduced nuclear condensation (%HG) although not with significant values. In the HQS fraction, an increase (*P* < 0.05) of the abnormality degree of chromatin structure (Alpha-T) was also noticed in LF bulls with respect to the HF bulls.

**Table 3:** *In vitro* sperm quality parameters assessed on frozen-thawed semen doses by CASA and FCM of 10 Holstein bulls based on fertility ranking in the CG and HQS fraction (LSM ± SEM).

**Fraction**	**CG**				**HQS Fraction**			
	**HF**		**LF**		**HF**		**LF**	
**Parameters**	**LSM**	**SEM**	**LSM**	**SEM**	**LSM**	**SEM**	**LSM**	**SEM**
MOT TOT	83.27^a^	4.00	63.02^b^	4.00	83.96	4.66	78.04	4.66
PROG	54.49^a^	3.07	43.80^b^	3.07	52.35	4.12	52.64	4.12
VSL	54.01	3.31	52.57	3.31	60.77	6.29	64.83	6.29
VCL	90.19	4.49	82.33	4.49	111.95	6.49	112.60	6.49
VAP	64.34	3.45	59.53	3.45	75.18	6.46	76.78	6.46
LIN	59.76	2.58	64.36	2.58	53.28	3.74	57.23	3.74
STR	83.73^a^	1.38	88.09^b^	1.30	79.51	1.97	84.11	1.97
WOB	71.16	2.13	72.85	2.13	66.27	3.13	67.73	3.13
ALH	2.96	0.14	2.73	0.14	3.69	0.18	3.65	0.18
BCF	9.85	0.38	10.25	0.38	8.48^a^	0.34	9.58^b^	0.34
VIAB	48.73	4.26	40.07	4.26	66.89	2.97	62.42	3.14
VIA	28.12	2.66	22.08	2.66	36.82	4.04	42.11	4.27
VDA	1.88	0.25	2.04	0.25	1.54	0.17	1.70	0.18
ALPHA-T	0.4097^a^	0.0016	0.4150^b^	0.0016	0.4025^a^	0.0020	0.4119^b^	0.0020
ATSD	0.0093	0.0009	0.0112	0.0009	0.0113	0.0006	0.0124	0.0006
%DFI	1.3337	0.3215	1.8830	0.3215	0.7327	0.2314	1.1106	0.2314
%HG	1.5775	0.2210	2.1160	0.2210	2.0627	0.1672	2.0502	0.1672

*MOT TOT, total motility; PROG, cells progressive motility; VSL, straight-line velocity; VCL, curvilinear velocity; VAP, average path velocity; LIN, linear coefficient; STR, straightness coefficient; WOB, wobble coefficient; ALH, amplitude of lateral head displacement, BCF, beat cross-frequency; VIAB, viable sperm; VIA, viable with intact acrosome; VDA, viable with disrupted acrosome; ALPHA-T, red/(red + green) fluorescence intensity; ATSD, Alpha-T standard deviation; %DFI, fragmented DNA sperm; %HG, high green fluorescence sperm*.

a,b *values within a row with different superscripts differ significantly at P < 0.05*.

A backward elimination analysis was performed to obtain a fertility prediction model between *in vitro* (sperm quality parameters) and *in vivo* (ERCR) fertility on both CG and HQS fraction. The initial model applied on both CG and HQS fraction was ERCR= MOT TOT + PROG + VSL + VCL + VAP + LIN + STR + WOB + ALH + BCF + VIAB + VIA + VDA + ALPHAT + ATSD + %DFI + %HG.

The analysis produced two final models that accounted for more than 78% of the variation of ERCR (CG: *R*^2^ = 0.88; HQS fraction: *R*^2^ = 0.78). The predictive equation of the final model on CG included eight variables (ERCR = ALPHAT + %DFI + ATSD + %HG + STR + VAP + VCL + WOB): four DNA integrity indicators (ALPHA-T: *F*-value 7.31, *P* = 0.0205, %DFI: *F*-value 31.65, *P* = 0.0002; ATSD: *F*-value 20.85, *P* = 0.0008; %HG: *F*-value 20.31, *P* = 0.0009), and four kinetic parameters (STR: *F-*value 15.27, *P* = 0.0024; VAP: *F*-value 12.54, *P* = 0.0046; VCL: *F*-value 12.09, *P* = 0.0052; WOB: *F*-value 10.01, *P* = 0.0090).

On HQS fraction, the predictive equation of the final included five variables (ERCR= ALPHAT + %DFI + MOT TOT + PROG + VSL): two DNA integrity indicators (ALPHA-T: *F*-value 43.07, *P* < 0.0001; %DFI: *F*-value 19.07, *P* = 0.0008), and three kinetic parameters (MOT: *F*-value 6.19, *P* = 0.0272, PROG: *F*-value 6.23, *P* = 0.0268; VSL: *F*-value 6.76, *P* = 0.0220).

**Figure 1:**
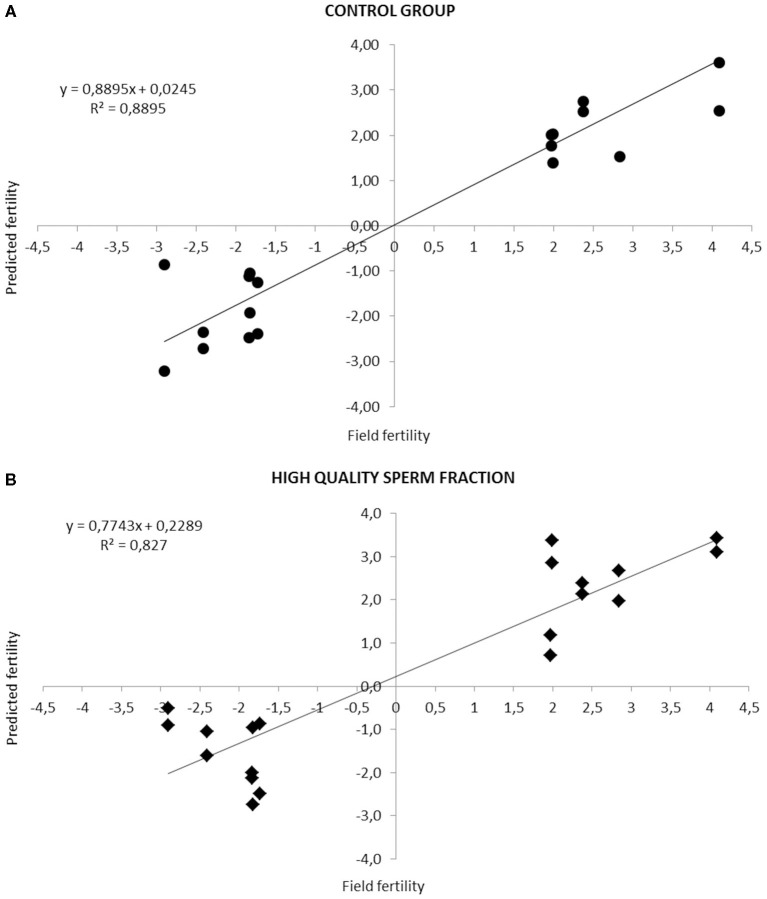
Relationship between fertility rates predicted by the equation based on the combination of different sperm quality parameters and the observed field fertility in CG **(A)** and HQS fraction **(B)**. The line shows the trend in the data.

Figure 1 highlights the high correlation (*R*^2^ = 0.88, Adjusted *R*^2^ = 0.8091; *P* < 0.05. *R*^2^ = 0.82, Adjusted *R*^2^ = 0.7062; *P* < 0.05) between real and predicted fertility found in CG and HQS fraction, respectively.

### Small RNA Sequencing, miRNA Identification and Profile Across HF and LF Bulls

**Figure 2:**
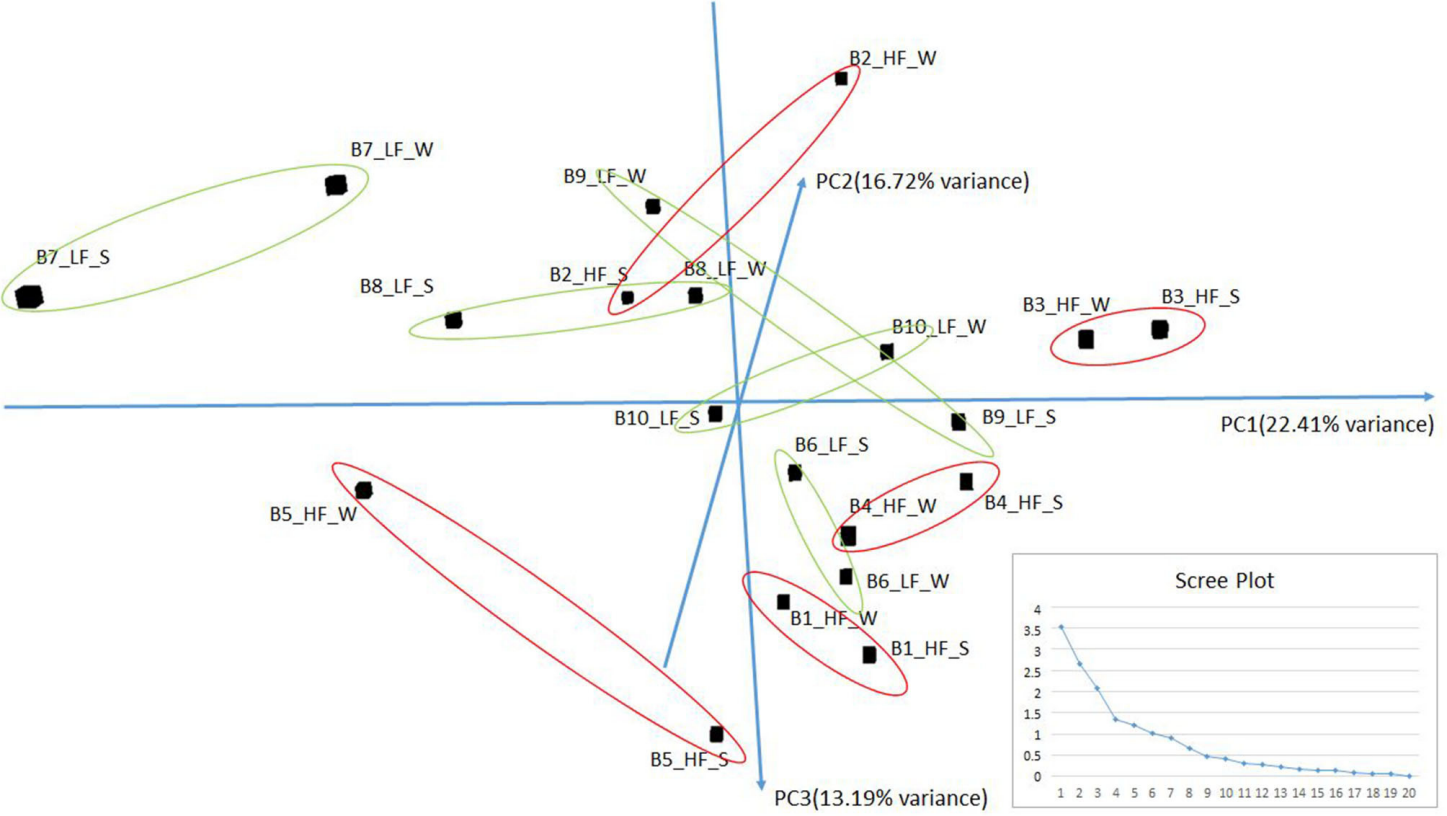
PCA of the more abundant miRNAs (*n* = 200) found in HF and LF bulls in the two seasons: Summer (S) and Winter (W). The scree plot, in the lower right corner of the figure, was reported to explore the optimal groups of the PCA (*x*-axis: principal component, *y*-axis: eigenvalues).

**Figure 3:**
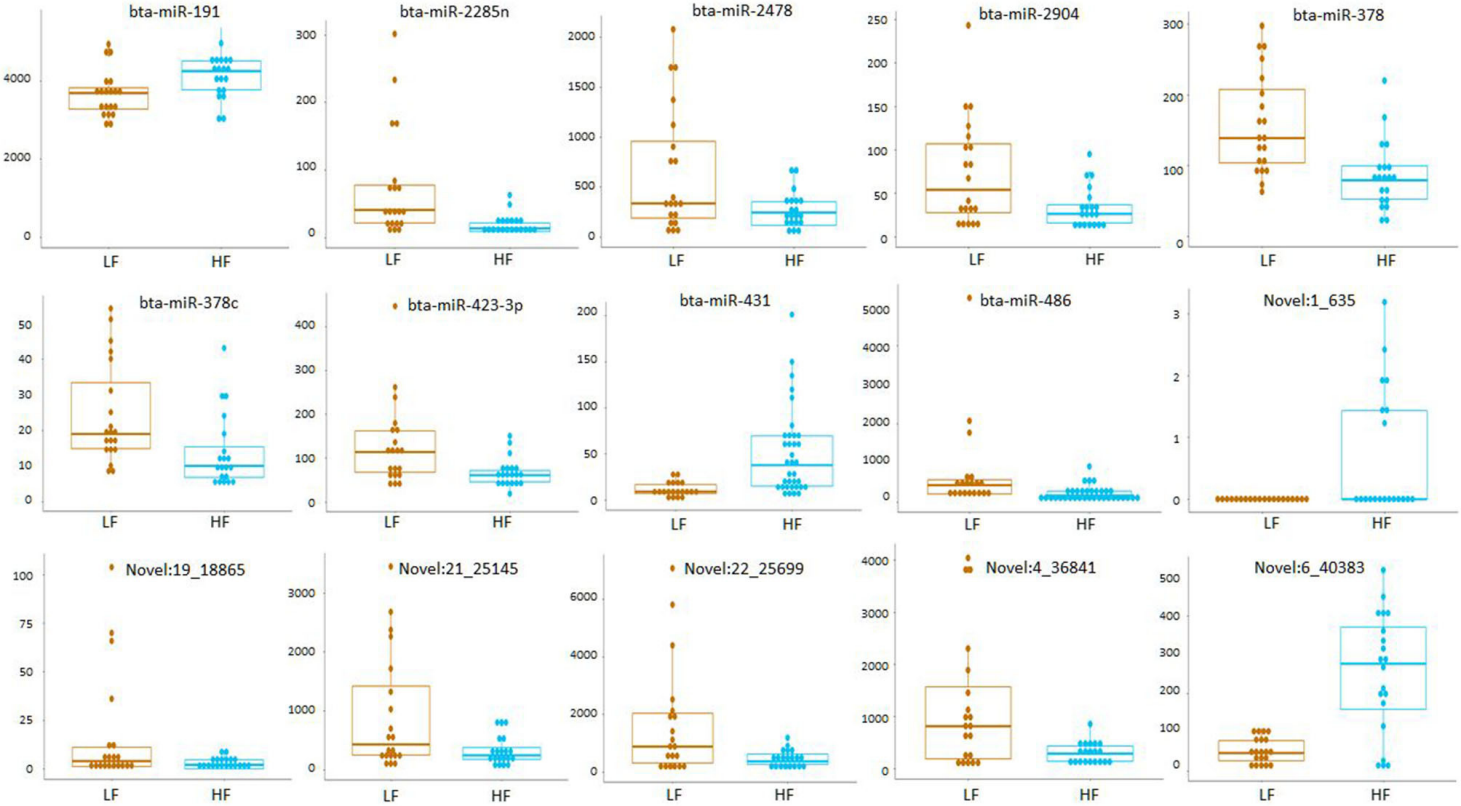
Read count of DE-miRNAs between HF and LF bulls.

Small RNA sequencing resulted in 145,180,999 reads after trimming and quality filtering with an average production of about 3.62 million reads per sample. An average of 632,000 miRNAs was detected by Mirdeep2 for each sample with the identifications of 923 unique miRNAs in total. Among these, 579 were known *Bos taurus* miRNAs, 98 were homologous of known miRNAs from other species, and 246 were new candidate miRNAs (Supplementary File 2). PCA performed on the 200 most abundant miRNAs grouped same animal closed together independently the season of semen collection and only partially separated HF (right lower corner) and LF (left upper corner) bulls (Figure 2). In fact, no significant variation was observed in miRNA expression from sperm collected in the two different seasons. On the contrary, comparison of HF and LF bulls results in 15 differentially expressed miRNAs (DE-miRNAs), nine of which were known, and the remaining were novel (Supplementary File 3). DE-miRNAs were prevalently highly expressed in the LF bulls (bta-miR-2285n, Novel:19_18865, Novel:4_36841, Novel:22_25699, Novel:21_25145, bta-miR-2478, bta-miR-2904, bta-miR-486, bta-miR-378c, bta-miR-378, bta-miR-423-3p) except for bta-miR-191, bta-miR-431, Novel:1_635 and Novel:6_40383, which show higher expression levels in the HF group (Figure 3).

### Possible Existing Association Between *in vitro* Semen Quality, Observed Fertility, and Differentially Expressed miRNAs

**Figure 4:**
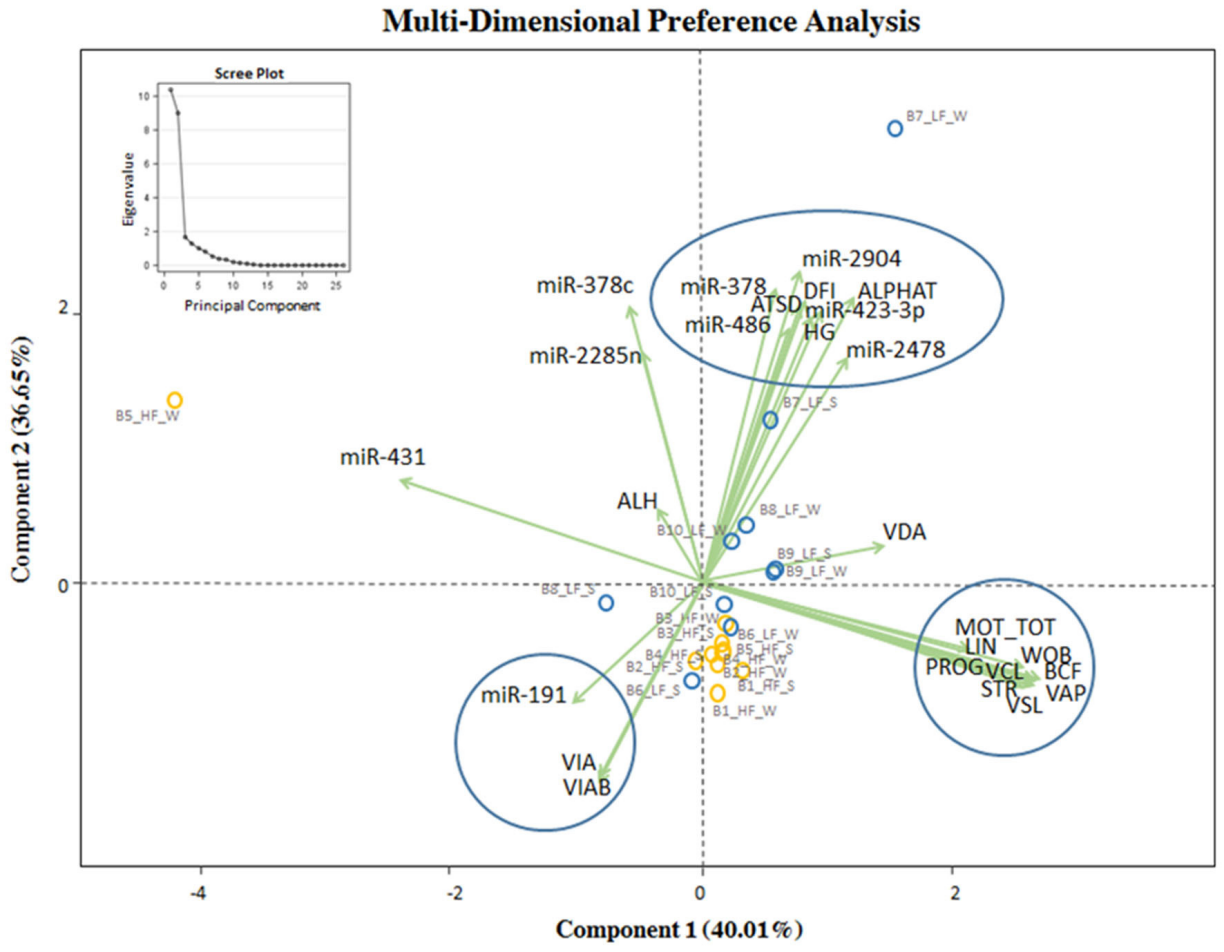
MDPREF analysis biplot based on the combination of *in vitro* sperm quality parameters, miRNA, and observed field fertility. The dots indicate the exact position of HF and LF bulls (yellow and blue, respectively) between the two components. The ellipse and circles represent the clustering relative to *in vitro* sperm quality parameters and miRNA: sperm motility and kinetics parameters (MOT TOT, MOT PROG, VCL, VAP, VSL, LIN, WOB, BCF, STR, on the right); DNA integrity and miRNA indicators (Alpha-T, ATSD, %DFI, %HG in the upper part); viability and acrosomal status and miRNA indicators (VIA, VIAB, in the lower part). The scree plot, in the upper left corner of the figure, was reported to explore the optimal groups of the PCA.

MDPREF was used to study the relationship between *in vitro* semen quality parameters and miRNA expression in relation to the observed field fertility (Figure 4). In the model, most of the variability is reached with the first two components (~76%). On the biplot, the trait scores are joined to the origin by the trait vector. The analysis produced two dimensions that seem to separate bulls according to their level of fertility (LF, HF), with the HF group more precisely clustered. Considering both *in vitro* sperm quality and miRNA expression, the first two components separated the vectors in three specific clusters, one relative to sperm motility and kinetics parameters (MOT TOT, MOT PROG, VCL, VAP, VSL, LIN, WOB, BCF, STR), the second one relative to DNA integrity indicators (Alpha-T, ATSD, %DFI, %HG), and the last one relative to sperm viability indicators (VIAB, VIA). The DNA integrity indicators (considered as damage) showed a positive relation with the group of bulls with LF, indicating also a positive relation with bta-miR-378c, bta-miR-423-3p, and bta-miR-486. Sperm viability indicators showed a positive relation with the group of HF bulls and miR-191. In Supplementary File 4 the correlation matrix of the Multi-Dimensional Preference analysis, showing the correlation between sperm quality and miRNAs, and the equation with select sperm quality parameters and miRNAs.

## Discussion

The current study was conducted with the aim to identify semen quality and miRNA biomarkers with the final goal to predict the fertility status in bulls. To categorize the most suitable parameters and provide a reliable prediction of bull fertility (among HF and LF bulls), we combined several *in vitro* sperm characteristics, assessed with advanced techniques such as CASA and FCM as well as miRNA expression by small RNA-sequencing with field fertility (ERCR).

In our study, the HQS fraction was successfully fractioned after Percoll gradient, showing good reproducibility between technical replicates and attenuating sample variabilities (20, 21). The improved HQS fraction was characterized by sperm with significant faster motion characteristics, more vital, with a better condition of the membrane integrity with respect to the CG. Similar results were observed for motility and membrane integrity in our previous study (7) and by Arias et al. (22). Although the average low levels of DNA damage shown were below the threshold for subfertility, in the HQS fraction, we observed a significant reduction of three variables out of four descriptive of DNA damage in contrast with Arias et al. (22) and Castro et al. (23), who did not find any significant difference for chromatin deficiency after Percoll gradient selection.

As reproductive success depends on the ability of the sperm cell to transfer information to the developing embryo, we analyzed many variables strictly related to sperm functions. According to our results, in the CG group, descriptors such as total and progressive motility were significantly higher in bulls with superior fertility. These results are in agreement with Gliozzi et al. (5) and in contrast to Kumaresan et al. (6) and Garcías-Macías et al. (24) who did not observed any significant differences in bulls' kinetic parameters between fertility groups. Motility is the most commonly used parameter in routine assessments performed in AI centers, but its correlation with fertility is not universally accepted (5), partially due to differences in estimation methods, CASA settings, thawing temperature, and media used for dilution (25). To overcome this problem and to be sure to evaluate the potential real fraction involved in sperm fertilization (in terms of semen quality and miRNA expression), we isolated the HQS fraction of cryopreserved semen. In the CG, other variables discriminated bulls according to their fertility level, such as the abnormality degree of chromatin structure (Alpha-T), in a significant way, and viable sperm (VIAB) and intact acrosome (VIA) as demonstrated by other studies for viability (5, 6, 26), acrosome integrity (4), and DNA (5, 6, 27), confirming the importance of these evaluations strictly correlated with sperm capacitation, *in vivo* fertilization, and early embryo development. Considering the HQS fraction, we observed that the Alpha-T variable can significantly discriminate among bull fertility levels, showing a lower DNA damage in bulls with superior fertility. In this group also total motility, viability, and %DFI have shown better values although not with statistically significant values.

In literature, high accuracy levels of the models for bull fertility prediction were achieved, such as reported in our previous study (5) and by Kumaresan et al. (6), both around an efficiency of *R*^2^ = 0.83–0.84. Our model developed in an earlier study (5), which provided the stepwise backward selection procedure application, has allowed us to reach, in this study, a major accuracy level equal to *R*^2^ = 0.88 in the CG and *R*^2^ = 0.82 in the HQS fraction. In both cases, the models include variables relative to kinetic parameters and DNA integrity indicators, accounting for most of the differences observed in bull fertility. Sperm motility traits and parameters related to chromatin structure were previously recognized as variables highly related with fertility and useful in the development of fertility predictive models (5, 28, 29). To our knowledge only Waterhouse et al. (30) and Gliozzi et al. (5) apply the stepwise backward selection procedures with the aim to develop models that associate *in vitro* semen quality with fertility prediction. Among the numerous variables evaluated, they found that only sperm motility traits and parameters related to chromatin structure are associated with field fertility, in agreement with our results, confirming that this model could be easily implemented in bull AI centers, which routinely evaluate sperm motility and may instruct external laboratories to assess DNA integrity. Recently, Alves et al. (31) reviewed the important role of miRNAs in physiological processes related to spermatozoa biogenesis, maturation, oocyte fecundation, and embryo development.

In cattle, different studies reported miRNA profile alteration associated with different sperm quality levels and fertility phenotypes. A previous study by qPCR miRNA profiling with a customized panel of bovine-specific miRNAs reported several miRNAs altered in HF and LF bulls isolated directly from cryopreserved semen (1, 10) or after purification with Percoll separation (8). Small-RNA sequencing of high and low motile fractions isolated from cryopreserved sperm using Percoll gradient, together showed variation of quality parameters and significant changes in miRNA expression (7). In this work, we characterized the miRNA content in the HQS fraction of HF and LF bulls collected in two different seasons. The relative expression of the most expressed miRNAs clearly shows that miRNA expression is strictly related to the animal and is not influenced by the season of collection. Although this is the first study reporting an individual-specific bull miRNA expression, the small non-coding RNA repertoire was recently reported to be different across cattle breeds and probably associated to animal genotypes (4).

HF and LF bulls present a subset of DE-miRNAs. Among 9 *Bos taurus* DE-miRNAs (miR-2285n, miR-378, miR-423-3p, miR-19, miR-2904, miR-378c, miR-431, miR-486, miR-2478), miR-2285n, miR-378 and miR-486 have already been proven to change their expression levels between low and high motile sperm populations in our previous study (7). The highest expression of miR-2285n and miR-486 in the semen of LF bulls was in agreement with the highest level of those miRNAs in the low motile fraction. MiR-486 expression in sperm was observed to change during sperm maturation in semen isolated from caput/caudal regions in mouse (32). MiR-486 function in controlling spermatogonial stem cell stemness gene expression and growth properties with a potential role in spermatogenesis and male fertility was recently described in mice (33). On the contrary, miR-378 observed to be overexpressed in the high motile fraction was upregulated in the LF bulls. In cattle, mir-378 was previously reported to be indirectly associated with semen quality (34). Single nucleotide polymorphisms in the inner centromere protein potentially affecting spermatogenesis and normal morphological characteristics of Holstein bull sperm was reported to influence the binding capacity to bta-miR-378 (34). Within other DE-miRNAs found in our study, miR-423-3p, highly expressed in the LF group, was previously detected to be positively correlated with the severity of asthenozoospermia in human sperm (35). Interestingly, within the only two miRNA positively associated with HF bulls, miR-191 showed a strong positive correlation with the fertilization rate of blastocysts (FR), in human *in vitro* fertilization experiments (36).

The MDPREF model, applied in this study to evaluate the relationship between *in vitro* semen quality parameters, miRNA expression on frozen-thawed motile sperm fraction, and observed bull fertility, seems to be able to discriminate bulls according to their level of fertility with more precision for the HF bulls. Semen quality variables are clustered in three specific groups, relative to sperm motility and kinetics parameters, to DNA integrity indicators, and to sperm viability indicators.

In our model, variables relative to sperm motility and kinematics have not shown any association between miRNAs and observed bull fertility, and a positive relation was revealed among sperm viability and DNA integrity indicators, miRNA expression, and bull fertility.

The DNA integrity indicators, which are descriptive of damage, positively correlate with LF bulls as well as with some miRNAs (miR-378, miR-486, miR-2285) previously reported to be differentially expressed between the low and high motile sperm fractions (7). However, no data concerning parameters related to DNA integrity was previously reported (7). Bulls of superior fertility correlate also with superior sperm viability indicators, in terms of viable sperm and viable sperm with intact acrosome and with miR-191 levels. Previous investigations on mir-191-5p expression related to sperm quality parameters show a significant correlation with sperm morphology but not with sperm density and viability in humans (36).

## Conclusion

In this study, we provide a reliable model to predict bull fertility, easily implementable in bull AI centers, which routinely evaluate sperm motility and may instruct external laboratories to assess DNA integrity, confirming the relationship between bull *in vitro* semen quality and *in vivo* fertility. Through an advanced semen quality assessment, which combines kinetic semen parameters originating from the CASA system and DNA analysis based on FCM, supported by statistical models, it was possible to establish that semen from LF bulls differs from that of HF individuals mainly in terms of sperm DNA integrity. Also viability and acrosome integrity concur to discriminate bulls according their fertility level.

We investigated differentially expressed miRNAs in the high-quality sperm fraction of bulls as candidate markers for evaluating fertility status. Fifteen miRNAs were differentially expressed in bulls with contrasting fertility. With our study, we applied a first statistical model integrating the relative expression of known DE-miRNAs and semen *in vitro* quality parameters to propose a solution to predict fertility that potentially could be used for further studies in the field in a wider population. As a novelty, the application of the MDPREF analysis demonstrated that a positive correlation was present among specific *in vitro* quality parameters (as motility viability and DNA integrity indicators), specific miRNAs, and bull fertility status.

Further studies are needed to evaluate these specific miRNAs as qualitative biomarkers for the prediction of assessment of sperm quality and the prediction of bull fertility. The integrated approach between advanced semen quality analysis and miRNA profiling could provide a model for prediction of fertility that might be beneficial to AI centers in the selection of bulls and facilitate the elimination of subfertile subjects or ejaculates with low fertilizing capacity.

## Data Availability Statement

The datasets presented in this study can be found in online repositories. The names of the repository/repositories and accession number(s) can be found below: the Sequence Read Archive (SRA) database, on NCBI server, and accession number: PRJNA727990.

## Ethics Statement

Ethical review and approval was not required for the animal study because the commercial frozen semen doses used in this study were acquired from an approved commercial artificial insemination bull centers.

## Author Contributions

EC, FP, FT, and AS conceived the study. FT isolated the spermatozoa fractions through Percoll gradient and evaluated sperm characteristics and carried out the statistical analysis. EC and PC performed the RNA extraction, libraries preparation and sequencing. BL carried out the bioinformatic analysis. EC carried out pathway analysis. FP acquired the funding and supervised the project. EC and FT wrote the manuscript and generated the figures. All authors read and approved the final manuscript.

## Conflict of Interest

The authors declare that the research was conducted in the absence of any commercial or financial relationships that could be construed as a potential conflict of interest.

## Supplementary Material

Supplementary Table 1 Effect of technical replicate on sperm quality variables assessed after Percoll separation in the High Quality Sperm Fraction (HQS). (LSM ± SEM).

Supplementary File 1 Example of Eukaryote Total RNA Pico profile obtained with Agilent Bionalizer for the sample B9_LF_S.

Supplementary File 2 Total raw and normalized counts for miRNAs expressed in sperm in HF and LF bulls and different seasons, including technical replicates.

Supplementary File 3 edgeR output for differential expression analysis between HF and LF bulls and different seasons.

Supplementary File 4 Correlation matrix of the Multi-Dimensional Preference analysis.

Supplementary File 5 Novel miRNA precursors sequences identified in High Fertility (HF) and Low Fertility (LF) bulls.

Supplementary File 6 Novel miRNA mature sequences identified in High Fertility (HF) and Low Fertility (LF) bulls.
