# Supplementary material for: A Combined Flow Cytometric Semen Analysis and miRNA Profiling as a Tool to Discriminate Between High- and Low-Fertility Bulls
Source: Front Vet Sci. 2021 Jul 19;8:703101. doi: 10.3389/fvets.2021.703101 (PMC8329915; doi:10.3389/fvets.2021.703101)
Supplement: Supplementary Table 1 — Effect of technical replicate on sperm quality variables assessed after Percoll separation in the High Quality Sperm Fraction (HQS). (LSM ± SEM). [file Table_1.docx]

**Supplementary Table 1. Effect of technical replicate on s**perm quality variables assessed after Percoll separation in the High Quality Sperm Fraction (HQS). (LSM ± SEM).

| **Sperm parameters** | **Technical Replicate A** | |  | **Technical Replicate B** | | **Technical Replicate**  **Effect** |
| --- | --- | --- | --- | --- | --- | --- |
|  | **LSQ** | **SEM** |  | **LSQ** | **SEM** |  |
| MOT TOT | 81,000 | 2,690 |  | 81,053 | 2,690 | ns |
| PROG | 52,322 | 2,016 |  | 52,677 | 2,016 | ns |
| VSL | 62,466 | 3,069 |  | 63,144 | 3,069 | ns |
| VCL | 113,257 | 4,428 |  | 111,300 | 4,428 | ns |
| VAP | 76,035 | 3,378 |  | 75,937 | 3,378 | ns |
| LIN | 54,896 | 2,068 |  | 55,622 | 2,068 | ns |
| STR | 81,722 | 1,551 |  | 81,904 | 1,551 | ns |
| WOB | 66,685 | 1,742 |  | 67,320 | 1,742 | ns |
| ALH | 3,724 | 0,138 |  | 3,621 | 0,138 | ns |
| BCF | 8,982 | 0,426 |  | 9,089 | 0,426 | ns |
| VIAB | 65,743 | 2,207 |  | 63,332 | 2,207 | ns |
| VIA | 38,373 | 1,695 |  | 39,948 | 1,782 | ns |
| VDA | 1,509 | 0,159 |  | 1,727 | 0,168 | ns |
| ALPHAT | 0,40973 | 0,00147 |  | 0,41373 | 0,00147 | ns |
| ATSD | 0,01138 | 0,00052 |  | 0,01276 | 0,00052 | ns |
| %DFI | 0,96200 | 0,26508 |  | 0,88138 | 0,26508 | ns |
| %HG | 2,19250 | 0,23945 |  | 1,99820 | 0,23945 | ns |

MOT TOT= total motility; PRG= cells progressive motility; VSL= straight-line velocity; VCL= curvilinear velocity; VAP= average path velocity; LIN=linear coefficient; STR= straightness coefficient; WOB= wobble coefficient; ALH = amplitude of lateral head displacement, BCF = beat cross-frequency; VIAB=viable sperm; VIA=viable with intact acrosome; VDA=viable with disrupted acrosome; ALPHA-T=red/(red+ green) fluorescence intensity; ATSD=Alpha-T

standard deviation; %DFI=fragmented DNA sperm; %HG=high green fluorescence sperm.
