# Supplementary material for: A Combined Flow Cytometric Semen Analysis and miRNA Profiling as a Tool to Discriminate Between High- and Low-Fertility Bulls
Source: Front Vet Sci. 2021 Jul 19;8:703101. doi: 10.3389/fvets.2021.703101 (PMC8329915; doi:10.3389/fvets.2021.703101)
Supplement: Supplementary File 1 — Example of Eukaryote Total RNA Pico profile obtained with Agilent Bionalizer for the sample B9_LF_S. [file Data_Sheet_1.docx]

**Supplementary File 1.** Example of Eukaryote Total RNA Pico profile obtained with Agilent Bionalizer for the sample B9_LF_S.
